# Sustainable pork production and processing: a step toward empowering tribal women in Northeast India

**DOI:** 10.3389/fnut.2024.1425020

**Published:** 2024-08-23

**Authors:** Shivani Mehta, Mahua Bhattacharjee

**Affiliations:** ^1^Manav Rachna International Institute of Research and Studies (MRIIRS), Faridabad, Haryana, India; ^2^Amity School of Economics, Amity University, Noida, Uttar Pradesh, India

**Keywords:** waste management, pork, sustainable development, India, value chain

## Abstract

This study explores the transformative potential of sustainable pork production and processing as a tool to empower tribal women in the northeast region (NER) of India. The NER is faced with multiple challenges, such as poverty, gender inequality, and poor livelihood methods. Therefore, enhancing sustainable production and processing methods for pork, which is their staple food, presents not only an opportunity for the socio-economic development of the region but also an effective tool for the economic empowerment of tribal women. Through a primary survey of pork value chain actors in Assam, Meghalaya, and Nagaland (the largest producers and consumers of pork in the NER), the study outlines the current practices and barriers to sustainable pork production methods. Although the consumption of pork has remained unchanged and that of processed pork items is on the rise, the production of pork is drastically declining. Therefore, reviving pork production in the NER can be instrumental in building sustainable livelihood models, especially for tribal women in the region. The study explores the effectiveness of a community-based, ‘model village approach,’ where capacity building around sustainable pork production, processing, and waste management techniques results in the economic empowerment of women. The findings from the post-impact analysis of the capacity-building approach call for policy intervention and the establishment of supportive networks to enhance the growth of a sustainable pork production system across NER, thereby contributing to the attainment of Sustainable Development Goal (SDG) targets proposed by the Indian economy.

## 1 Introduction

The northeastern region (NER) of India is characterized by diverse topography and a rich environment, making it suitable for crop cultivation and livestock farming factors that majorly contribute to over 90% of livelihood income in the region (1). Pig production and consumption are an integral part of the northeast Indian community. Although the demand for pork and its level of consumption across NER is largely unchanged and rather growing, the level of pig production is witnessing a sharp decline in the region (2). This decline in pig production is due to two major challenges that are faced by the locals: (a) the production process is largely unorganized and confined to backyard farming and (b) the challenges faced by pig farmers in effective waste management (3).

The mixed farming pattern widely used by households across NER creates a possibility of a mutually beneficial relationship where crop residue can act as feed for livestock and the manure from livestock can replenish the soil for crop production (4). This symbiotic relationship creates a possibility for sustainable production, which to date largely remains underutilized in the region. Furthermore, analysis of consumer preference in the region highlights that pork is not only the dominant form of meat consumption among households but also there is a rise in demand for processed pork items (1). This creates a great avenue for building sustainable livelihood models for rural tribal households where they can effectively raise their incomes (through processed pork production techniques) and lower the cost of agricultural produce (by engaging in effective waste management methods), thereby moving toward higher standards of living. However, for the successful implementation of new sustainable livelihood models, it is imperative to understand the local socio-economic and cultural contexts in the NER. In the states of Assam, Meghalaya, and Nagaland, tribal women play a central role in rearing pigs, managing household finances, and participating in local markets. However, they are not an active agent in the market chain or pork value chain (5). As a result, the monetary benefit derived from value addition fails to reach the women pig rearers, curtailing their empowerment.

This study, therefore, outlines some key findings from the NASF-ICAR-funded project (2019–2022) titled “Pork Marketing Chains in North East India for Sustainable Livelihood of Tribal Women (Assam, Meghalaya, and Nagaland).” Extensive primary research was conducted for 3 years, where the different market and value chain actors of pork were studied in Assam, Meghalaya, and Nagaland. The major agents identified across the value chain were producers (largely tribal women pig rearers), traders/middlemen (largely men, who would take the pig to urban markets), retailers, and consumers. A random sample of 1,000 producers, 50 traders, and 250 consumers from each state was collected to identify the recent trends and challenges in pork production and consumption in the region. Quantitative tools, such as regression analysis and PCA, were applied alongside qualitative analysis to not only identify the challenges but also examine the role of sustainable pork processing and waste management systems as a means to empower tribal women in the region.

Therefore, this study outlines the transformative potential of sustainable pork production and processing as a means to empower tribal women in the NER of India. The study examines the pork value chain and finds that high feed cost, expensive pig stock, lack of transportation facilities, problems pertaining to bio-security, and poor veterinary services are among the main root causes for lowered pig production in the region. On the contrary, consumer analysis signifies that pig consumption continues to be a staple diet among the population, and more so, the demand for processed pork items, such as ham, nuggets, and sausages, is on the rise.

The study proposes a model for a community-based pork production system using a ‘model village approach’. The approach will hold significant importance in empowering women through sustainable pork production and processing methods, which can lead to broader socio-economic benefits including food security and gender equality (6). The study calls for policy intervention, extensive capacity building, and the establishment of supportive networks to enhance the growth of the NER, thereby contributing to the attainment of SDG targets.

## 2 Context: present trends in pork production and processing

Pig production and processing is a viable source of sustainable livelihood for many small farmers, especially in tribal areas of the NER. Presently, the pig production methods adopted are largely confined to small-scale backyard farming (7). The tribal households engage in informal methods to produce pigs, which are largely used for local consumption rather than for market. As a result, the possible income generation from the commercialization of pig production is lost in the process (8).

**Figure 1:**
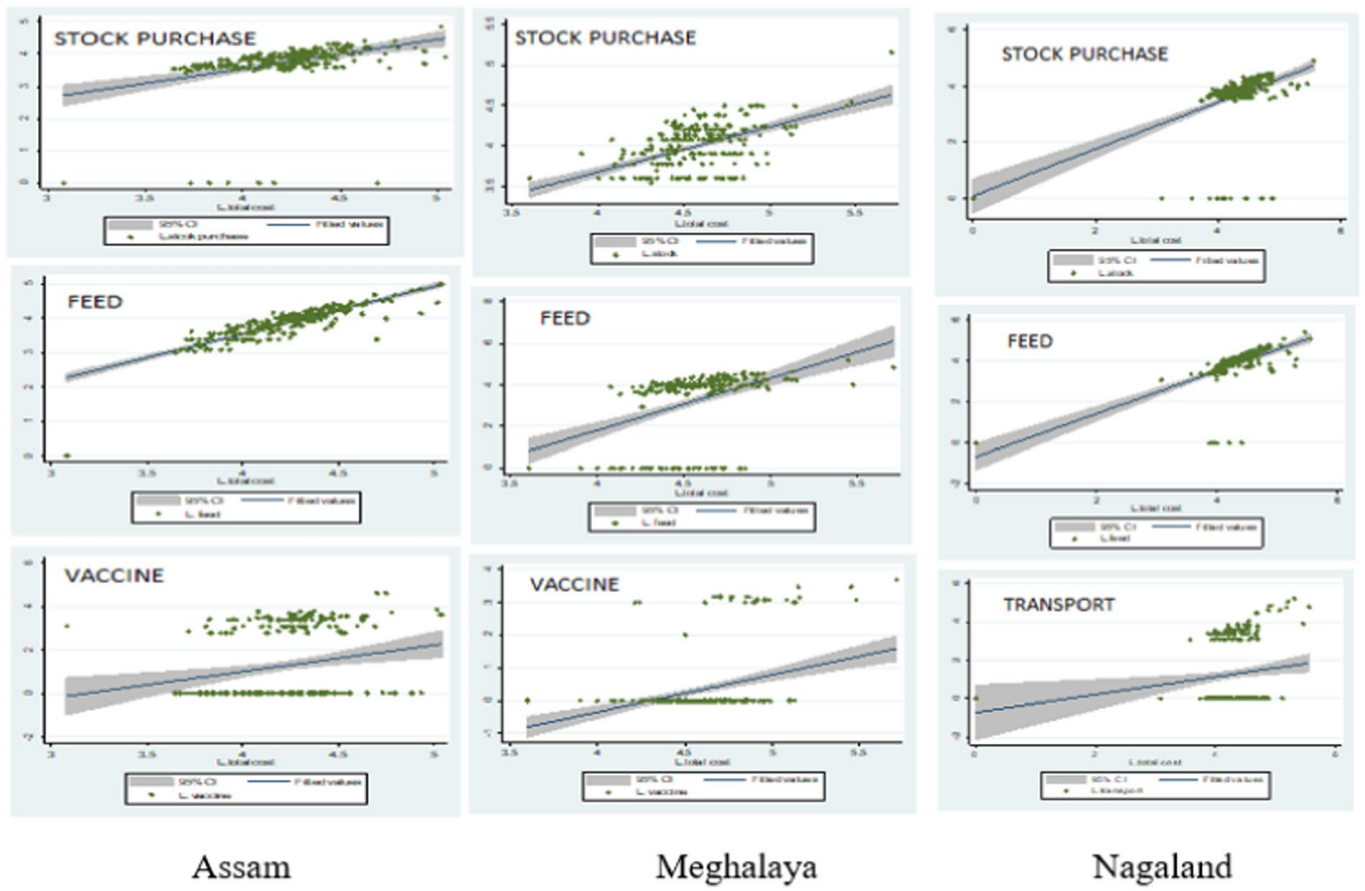
Major cost determinants curtailing pork production in the NER as per regression analysis performed on 1,000 producers from Assam, Meghalaya, and Nagaland each (the biggest states in terms of pork production and consumption in the NER). Reprinted with permission from International Journal of Agricultural and Statistics Sciences (9).

**Figure 2:**
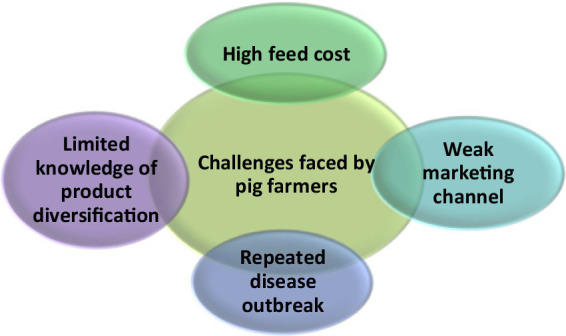
Other crucial factors lowering pig production across NER. Source: Compiled and constructed through the primary survey by the author under the NASF-ICAR-funded project.

The regression analysis performed (Figure 1) on 1,000 pig producers from Assam, Meghalaya, and Nagaland portrays that high feed cost, lack of vaccination facilities, high cost of stock purchase, and high transportation cost are the major factors that curtail the growth of pork production across NER (9). Furthermore, through focused group interviews of tribal women pig producers, the study finds that weak marketing channels and a lack of knowledge of product diversification also act as barriers to the development of pig production in the region (Figure 2). Irregular supply chains, limited participation in the marketing chain, and a less competitive credit market are some of the major challenges faced by small and marginal farmers under the unorganized structure of backyard pig farming.

The production and rearing of pigs across NER are dependent on locally available feed, which largely is sourced from local farm and kitchen residues (10). A lack of proper feed nutrients often results in low pig mass and less immunity among pig stock. Furthermore, lack of proper housing/shelter and bio-security measures exposes the available stock of pigs to adverse temperatures and flu which is a common cause for the loss of pig stock (11). Climate change and environmental factors, such as flooding and outbreaks of diseases such as African swine fever (ASF) or classical swine fever (CSF), cause serious disruptions in pig production, making it a less preferred occupation among rural households (Figure 2).

The above-mentioned factors collectively impede the growth of pig production in the northeastern states, despite the high demand for pork in the region. Infrastructural issues, such as inadequate veterinary services, poor transportation networks, and insufficient access to quality feed, further exacerbate the difficulties faced by tribal women pig farmers (12). Additionally, socio-cultural factors, including traditional farming practices and limited market access, restrict the adoption of modern breeding techniques and commercial farming methods, making pig rearing and production a less popular occupation among the growing youth population in the region. Addressing these challenges requires a holistic approach that integrates improved infrastructure, enhanced veterinary support, and targeted socio-economic interventions to support and modernize pig farming practices.

**Figure 3:**
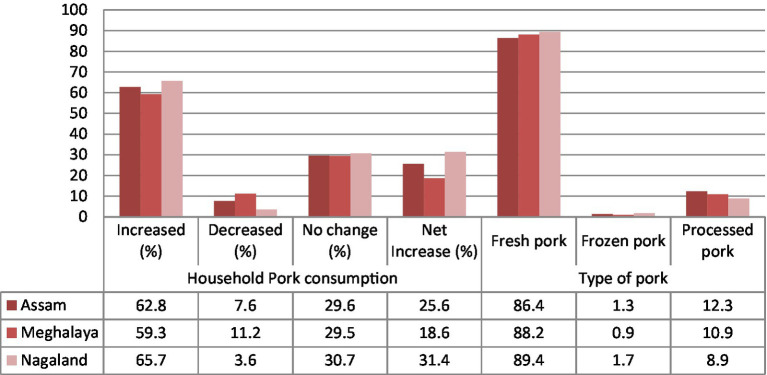
State-wise net increase in household pork consumption (net of marginal decline and no change depicted by households). Adapted with permission from the International Journal of Food and Nutritional Sciences (1).

Although the production of pig and indigenous pig farming methods is dwindling across NER, the consumption data collected from over 250 consumers in each of Assam, Meghalaya, and Nagaland depict a contrasting picture. The data collected through the primary survey show that the household consumption demand of fresh, frozen, and processed pork across NER has by and large increased (net of households where consumption has reduced marginally or has seen no change) over the years, as shown in Figure 3.

**Figure 4:**
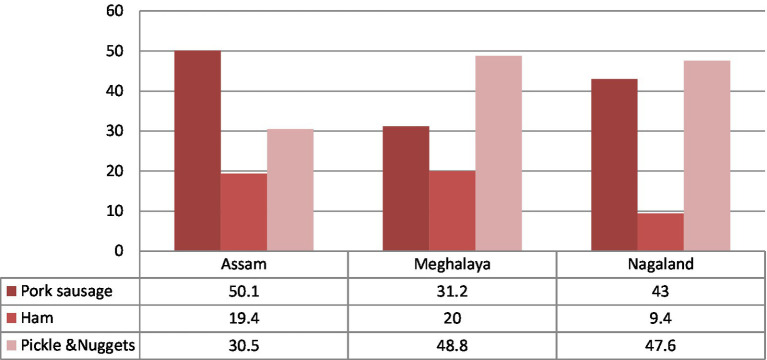
State-wise household preferences of pork consumption for processed pork items. Adapted from Bhattacharjee et al. (13), licensed under CC BY 4.0.

Furthermore, the study finds that consumers in the NER depict the highest preference for fresh pork, with the demand for processed pork items on a steady rise (Figures 3, 4). Households are increasingly becoming more open to the consumption of processed pork products such as ham, sausage, and nuggets. Furthermore, the study finds that people show a higher willingness to buy safer pork across all states (13).

As a result, the present production and consumption pattern of pork in the region clearly outlines a growing demand gap. The consumer analysis clearly shows that household demand for pork is either consistent or increasing along with the change in consumer preference toward processed pork items such as ham, sausages, pickles, and nuggets. As a result, growth in pig production holds significant potential to have a positive impact on the livelihood of millions of resource-poor, underprivileged, landless, and marginal farmers in the region.

## 3 Sustainable pork production and processing as a key to empower tribal women

**Figure 5:**
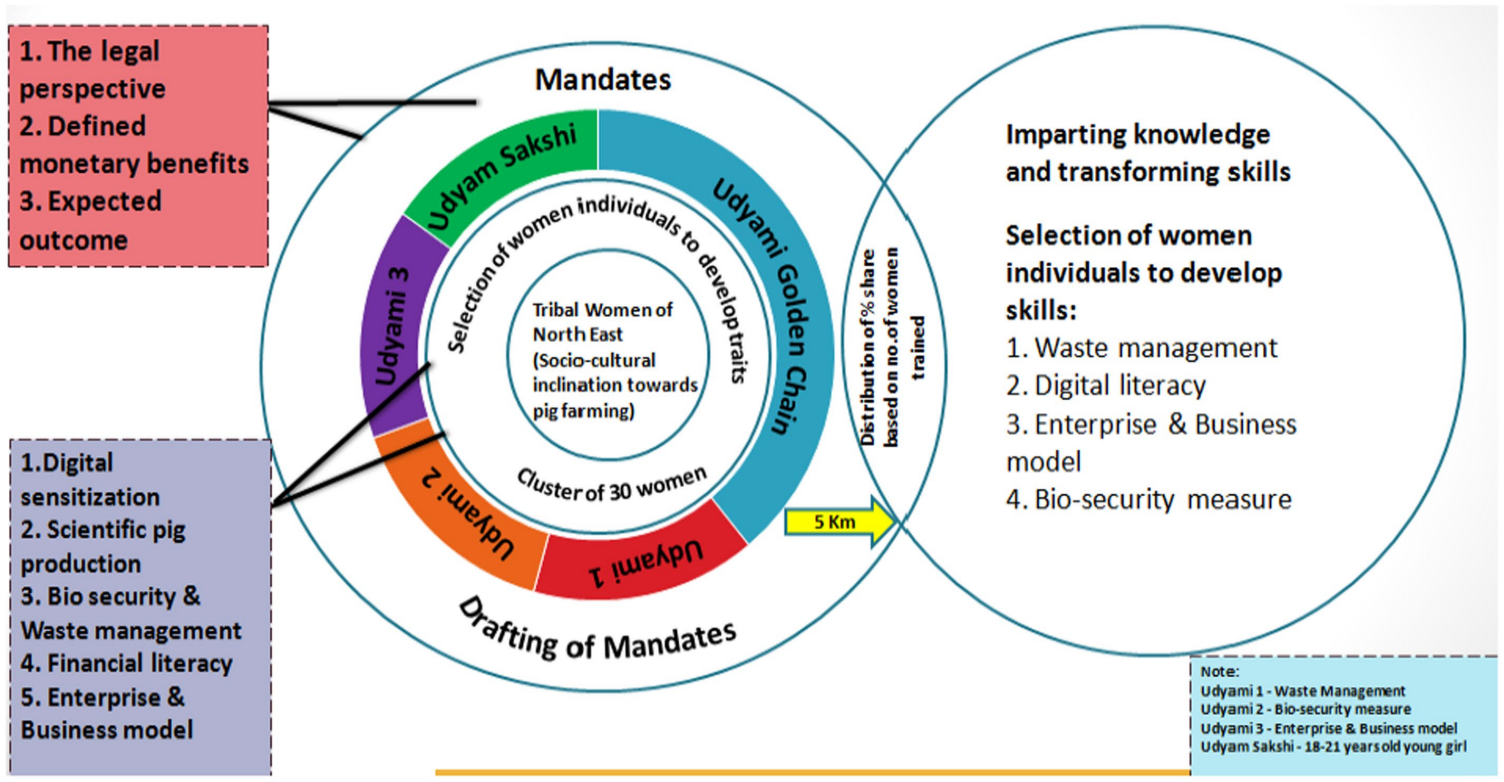
Framework to economically empower tribal women of the NER. Source: Compiled and constructed through impact analysis by the author under the NASF-ICAR-funded project.

There are two main obstacles faced by households (largely led by women) that engage in pig production. The first issue that women farmers face is the disorganized nature of farming, which makes it challenging for them to keep an eye on the productivity of their animal stock. Low productivity further keeps income generation at a subsistence level, pushing the pig farmers into the vicious circle of poverty. Second, the effective handling of animal waste is an extremely challenging task that is often poorly managed across all households (3). However, the study proposes that by applying the following framework, as shown in Figure 5, these obstacles can be converted into strengths for tribal women across NER and can be instrumental in economically empowering them.

The study proposes the applied and tested cluster-based ‘model village approach’, where trainings and extensive capacity building on a pre-identified women’s cluster were performed (14). A cluster of 30 tribal women engaged in pig production was identified from each region and was extended trainings for their skill development. These trainings were delivered through the first-ever concept of the ‘Piggery Field School’, where these women were taught effective ways to develop home-based feed through ‘Silage’ making, bio-security measures to curb the loss of pig stock, simple pork processing techniques, and waste management methods. These capacity-building techniques proved successful in enhancing their knowledge and skills, thereby acting as a source for substantially augmenting household incomes. The study proposes that these trained women, referred to as ‘Udyamis’, then act as knowledge disseminators to commercially transfer the skills to the tribal women with a socio-cultural inclination toward pig farming.

The study therefore dwells deeper into the two emerging prospects where such training and capacity building can help double rural household incomes: (a) sustainable pig production through effective waste management and (b) product diversification toward processed pork products. Developing capabilities of tribal women in these two areas can first help revive pig production across NER and, second, economically empower tribal women in the region.

### 3.1 Sustainable pig production through effective waste management

**Figure 6:**
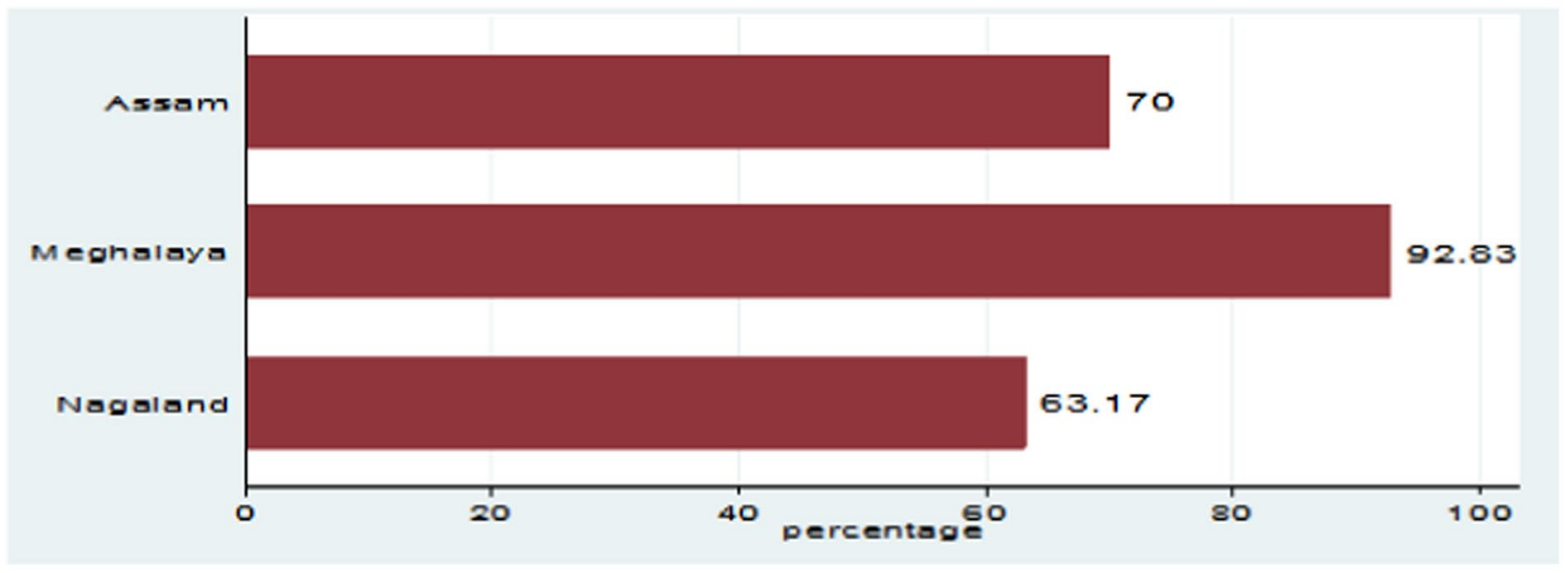
Pig waste collection by the percentage of the household. Source: Compiled and constructed through the primary survey by the author under the NASF-ICAR-funded project.

Some of the major challenges faced by pig farmers due to inadequate management of livestock waste released in backyard farming are because of information asymmetry regarding the scope of organic farming and biogas production in agriculture (7). The study finds that a negligible portion of the population disposes of pigs’ wastes scientifically, despite the fact that the majority of people in the northeast collect pig dung (Figure 6).

Although the collection of pig waste is found to be a common practice among rural households, its effective utilization is rather absent. A very small percentage of households, i.e., approximately 38.5% in Meghalaya and 32.3% in Nagaland, use their pig dung for agricultural purposes, which creates a big possibility to enhance rural household income through effective waste management initiatives (15).

The data for pig manure by content as outlined in Table 1 show that the conversion of ‘pig dung’ into ‘pig manure’ can be a fruitful avenue through which tribal women can earn subsidiary income. Pig manure is an excellent source of plant nutrients as it is rich in phosphorus, potassium, and magnesium. Therefore, converting the dung into manure and then selling this manure to other agricultural farmers can help tribal women earn additional income.

**Table 1:** Nutrient content in pig manure.

Manure	Phosphorus	Potassium	Calcium	Magnesium
Cow dung	0.43	0.53	1.82	1.94
Pig manure	3.3	0.4	1.28	1.3
Goat manure	0.9	1	2.17	0.93
Chicken manure	2	0.82	1.14	0.89

Source: Bhattacharjee et al. (1).

Therefore, the creation of Udmayi 1-tribal women who specialize in waste management techniques at the cluster level can help solve the problem of pig waste management on the one hand (making pig farming sustainable) and create avenues for additional income generation for tribal women on the other hand (through the sale of pig manure for agricultural purposes). Providing specialized training to Udmayi 1 on the collection of pig waste, its fermentation, drying, cooling, and the correct ratio of adding raw material for composting can prove instrumental in augmenting the income of tribal women.

### 3.2 Product diversification toward processed pork products

In the NER agricultural ecosystem, women portray active engagement in livestock farming and particularly piggery among the tribal communities. Engaging women in small-scale pig farming businesses can be a great way of utilizing family labor to earn additional income. Pig farming and, especially, pork processing hold enough scope for entrepreneurship development across NER. This growth in women’s entrepreneurial initiatives has further been linked to improved growth, increased wealth, and quality of life among tribal communities (16).

**Figure 7:**
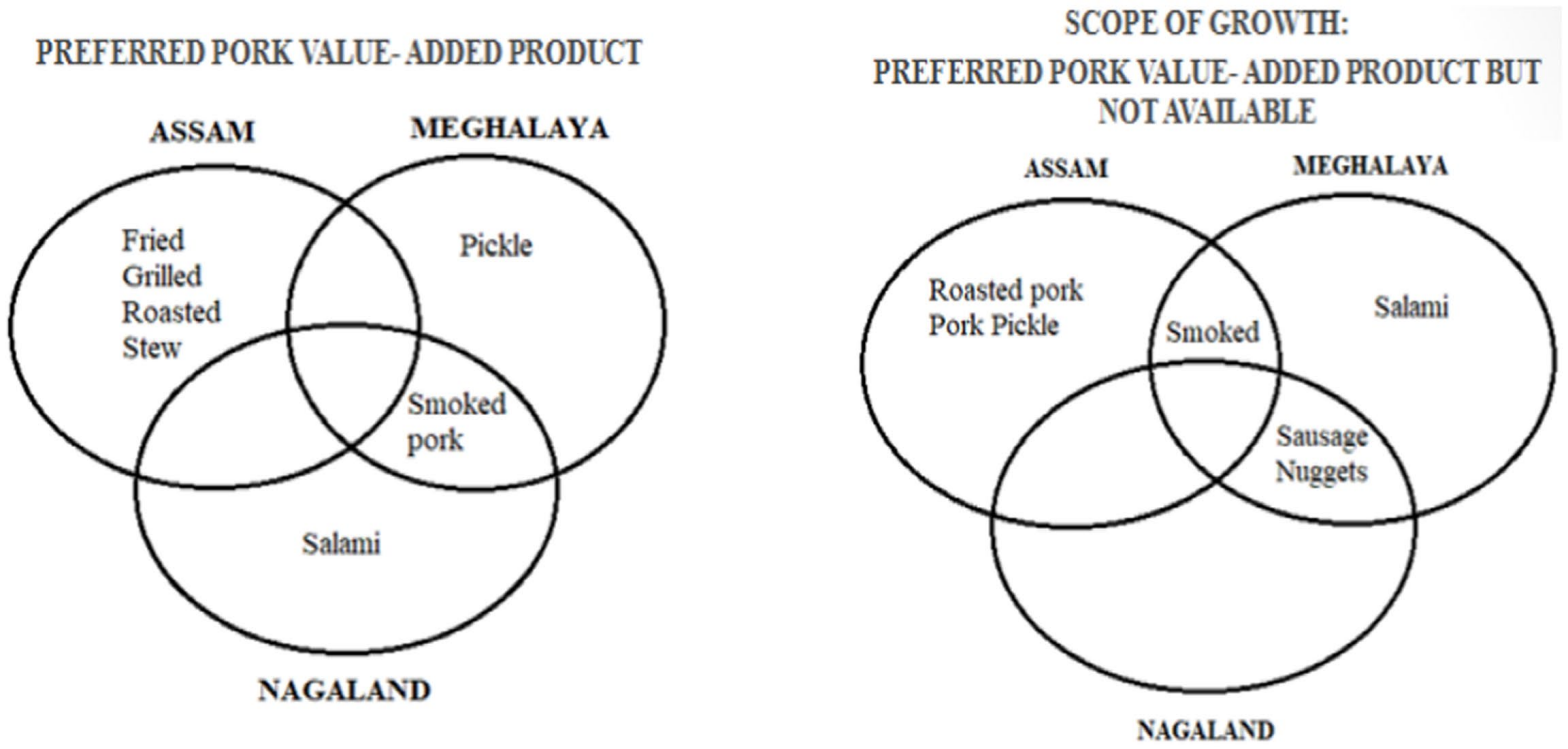
Preference of pork processed items as per their availability. Source: Compiled and constructed through the primary survey by the author under the NASF-ICAR-funded project.

In line with the growing consumer preference toward processed pork items (Figure 7), tribal women should be extended training or capacity building around meat processing. Simple pork processed items, such as pork sausages, patties, nuggets, kebabs, pickles, and pork soup, hold great potential for enhancing entrepreneurship among tribal women of the NER.

This processing of simple pork products can be initiated at the household or a cluster level without any huge capital investment. As a result, training Udmayi 3-tribal women of the NER in the processing of pork products can act as a major source of additional income and sustainable livelihood. Furthermore, the higher price of processed items, *viz.*, selling fresh pork, will help augment the standard of living among rural households.

Therefore, encouraging value addition and diversification within the pork marketing chain can enhance economic returns. Processing pork into various products, such as sausages, smoked pork, and packaged meat, can open up new market opportunities and increase profits. Providing women with training and resources for small-scale processing units can create additional income streams and reduce waste. Furthermore, exploring niche markets, such as organic or free-range pork, can cater to growing consumer preferences for ethically produced food.

The above-mentioned capacity-building initiatives for the creation of Udmayi 1—women specializing in waste management techniques—and Udmayi 3—women engaged in pork processing—were enacted in three districts, namely, Rangia (Assam), Ri Bhoi (Meghalaya), and Phek (Nagaland).

**Figure 8:**
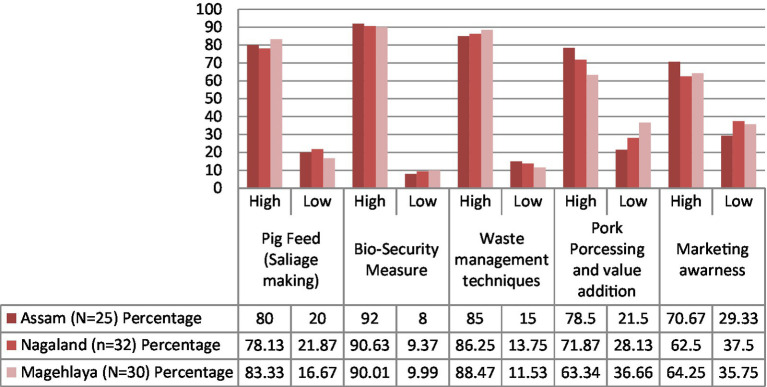
Findings of post-impact analysis through cluster-based ‘model village approach’. Source: Compiled and constructed through impact analysis by the author under the NASF-ICAR-funded project.

Post-impact analysis conducted among the women clusters after the capacity-building programs revealed that approximately 80% of tribal women added significant knowledge in areas pertaining to pig feed, bio-security measures, and waste management as shown in Figure 8. It was also found that trainings were extremely helpful in developing skills related to marketing awareness, pork processing, and value addition, but it also requires constant handholding and focused intervention to boost local entrepreneurial skills, especially at the nascent stages.

## 4 Conclusion

The study explored the potential of sustainable pork production and processing as a transformative force for the empowerment of tribal women in Northeast India. Through a detailed analysis of current trends, challenges, and opportunities, the study highlighted the significant role that these activities can play in improving the socio-economic status of tribal women, enhancing household income, and promoting environmental sustainability in the region. Our findings reveal that by integrating traditional knowledge with modern sustainable practices, tribal women can significantly contribute to and benefit from the pork value chain. This not only strengthens their economic independence but also ensures the preservation of their cultural heritage and the sustainable development of their communities (17).

The study emphasized the importance of supportive policies, capacity building, and the creation of cooperative models through cluster-based ‘model village approach’ as critical enablers for their active participation and leadership of women in this sector. Post Capacity building process, survey findings show that trainings around pork processing and waste management techniques have proved successful in significantly improving productivity and profitability among tribal women. Therefore, it is safe to assert that in the near future, promoting sustainable farming practices, such as integrated farming systems, organic feed, and waste management, shall be instrumental in ensuring long-term viability and environmental health in the region.

Strengthening pork marketing chains in Northeast India through innovative methodologies can transform the livelihoods of tribal women, fostering economic empowerment, and sustainability. By leveraging cooperative models, capacity building, technology, value addition, sustainable practices, and supportive policies, these communities can achieve greater economic security and social wellbeing. Tailoring these approaches to the unique cultural and socio-economic context of Assam, Meghalaya, and Nagaland will ensure their effectiveness and sustainability.

In conclusion, sustainable pork production and processing present a viable and impactful pathway toward the empowerment of tribal women in Northeast India. By harnessing the synergies between economic empowerment, cultural preservation, and environmental sustainability, this sector offers a model for holistic development that can be replicated in other contexts. As such, it calls for a collaborative effort among government bodies, non-governmental organizations, the private sector, and the communities themselves to realize the full potential of this initiative. Through concerted action and sustained commitment, we can move toward a future where tribal women are not only participants but leaders in creating a more sustainable and equitable world.

## Data availability statement

The raw data supporting the conclusions of this article will be made available by the authors, without undue reservation.

## Author contributions

SM: Conceptualization, Data curation, Formal analysis, Funding acquisition, Investigation, Methodology, Project administration, Resources, Supervision, Validation, Writing – original draft. MB: Conceptualization, Data curation, Formal analysis, Funding acquisition, Investigation, Methodology, Project administration, Resources, Supervision, Validation, Writing – review & editing.
